# The Australian Feeding Infants and Toddlers Study (OzFITS) 2021: Highlights and Future Directions

**DOI:** 10.3390/nu14204343

**Published:** 2022-10-17

**Authors:** Merryn J. Netting, Najma A. Moumin, Maria Makrides, Tim J. Green

**Affiliations:** 1 Discipline of Pediatrics, Faculty of Health and Medical Sciences, University of Adelaide, Adelaide, SA 5000, Australia; 2 SAHMRI Women and Kids Theme, South Australian Health and Medical Research Institute, Adelaide, SA 5000, Australia; 3 Nutrition Department, Women’s and Children’s Health Network, Adelaide, SA 5006, Australia

**Keywords:** infants, toddlers, dietary transition, Australia, nutrient intake, survey

## Abstract

The 2021 Australian Feeding Infants and Toddlers Study (OzFITS 2021) is the first nationwide survey of the feeding practices of children under 2 years. Key Findings: Nearly half of the infants were exclusively breastfed to 4 months, and breastfeeding duration was long, with 68% of infants breastfed to 6 months and 44% breastfed into their second year. Infants were introduced to complementary foods at the appropriate time, between 4 and 6 months. We found a mismatch between the number of recommended servings from each food group in the Australian Dietary Guidelines and the dietary intake of toddlers in our study. Toddlers consumed twice as many fruit servings as recommended, and nearly all consumed discretionary foods despite no allowance for these foods. While most toddlers consumed the recommended dairy serves, they consumed half the recommended servings for other food groups—meats and alternatives, grains, and vegetables. The modeling that informed the Australian Dietary Guidelines did not include an allowance for breastmilk; this needs to be addressed, as a quarter of toddlers in OzFITS 2021 received 30% or more energy from breastmilk. Infants and toddlers met their requirements for most nutrients. One exception was iron, where 90% of older infants and 25% of toddlers had inadequate intakes. Excessive sodium intake was also of concern, with 1 in 3 toddlers exceeding the upper limit of 1000 mg/day. Here, we discuss additional findings, study limitations, gaps in the evidence base, and future directions.

## 1. Introduction

During the first two years, an infant must transition from a milk-based diet to consuming foods from the family table. What a child is fed at this time is critical for optimal growth and development. Nutrient or energy deficits during this period may lead to impaired growth and cognitive development and increased disease risk [1,2]. Conversely, excess weight gain during the first two years is associated with overweight and obesity, which may persist into later life, increasing the risk of chronic disease [3]. Nutrient-dense foods are required to meet the young child’s high nutrient requirements relative to the amount of food consumed [4].

Despite the importance of early life feeding, there is little national data on contemporary infant and young child feeding practices in high-income countries. In Australia, we have national data on breastfeeding rates and duration and the timing of introduction to complementary foods [5]. However, there are no Australia-wide data on food and nutrient intakes in this population, nor information on the extent to which parents/caregivers follow national infant and toddler feeding guidelines. The Australian Feeding Infants and Toddlers Study 2021 (OzFITS 2021) addresses this gap by surveying parents/caregivers of 598 infants and 542 toddlers from all Australian states and territories [6]. Information on breastfeeding practices, the use of breastmilk substitutes, and the timing of solid food introduction, including food allergens, was captured. Moreover, we included a 24 h dietary intake record to estimate nutrient intakes and the prevalence of inadequate and excessive nutrient intakes in young children.

## 2. Summary of Key Findings and Implications

### 2.1. Breastfeeding, Breast Milk Substitutes, and the Introduction of Complementary Foods

The Australian Infant Feeding Guidelines [7] recommend exclusive breastfeeding for around six months, followed by the introduction of nutritious complementary foods with continued breastfeeding for 12 months and beyond. In Netting et al. [8], we report a high initiation rate and a long duration of breastfeeding, with 68% of infants breastfed to 6 months and 44% breastfed into their second year. Nearly half of the infants were exclusively breastfed to 4 months, and one-third of infants were given breastmilk substitutes. Of concern was that parents frequently reported that their child’s first exposure to breastmilk substitutes was in the hospital soon after birth. Interestingly, many mothers resumed breastfeeding their infants until complementary foods were introduced. Early exposure and subsequent cessation of breast milk substitutes has been associated with an increased risk of cow’s milk allergy [9].

Very few infants were fed solid foods before four months, which is encouraging as the early introduction of complementary foods may increase the risk of obesity, gastrointestinal disorders, and food allergy [7,10]. Likewise, delayed introduction to complementary foods is associated with feeding difficulties, growth faltering, and micronutrient deficiencies [7].

### 2.2. Introduction to Common Food Allergens

Recommendations for infant feeding specific to allergy prevention have recently changed based on new high-level evidence supporting early and regular exposure to common food allergens to reduce the risk of developing IgE-mediated food allergy [11,12]. The Australasian Society of Clinical Immunology and Allergy updated its advice in 2016 to reflect this new evidence, which has been widely promoted by the federally funded National Allergy Strategy allergy prevention campaign [13]. Parents enrolled in OzFITS 2021 followed this advice, with 97% introducing eggs and 94% introducing peanuts by one year; however, we did not capture the frequency of exposure in this study. Caregivers are also advised to give their infant potential food allergens regularly (twice weekly) once introduced to prevent food allergies from developing [13]. In a South Australian study [14], we found most caregivers introduced allergens by 1 year, but many were not regularly exposing their infant to potential allergens.

### 2.3. Foods and Drinks

By 12 months, children should have transitioned from a milk-based diet to consuming nutritious family foods consistent with the Australian Dietary Guidelines [7,15]. OzFITS 2021 is the first study to compare the dietary intakes of Australian toddlers to the recommended servings of all five food groups (fruits, vegetables, dairy cereals and grains, and meat and meat alternatives) [16]. We found a mismatch between the number of recommended servings from each food group and the dietary intake of toddlers in our study [15,17]. Toddlers consumed twice as many fruit servings as recommended, and nearly all toddlers consumed discretionary foods, despite no allowance for these foods. Consumption of fruit and discretionary foods may have displaced other food groups. While most toddlers consumed the recommended dairy serves, they consumed half the recommended serves for other food groups.

Infant feeding guidelines recommend that breastfeeding be continued until 12 months of age and beyond, for as long as the mother and child desire [7]; however, breastmilk is not included in the dietary modeling underpinning the recommended dietary pattern for toddlers [15,16]. In our study, 44% of toddlers were still breastfed, and one-quarter received nearly one-third of their daily energy intake from breastmilk. Because of the high prevalence of continued breastfeeding, we stratified our analysis by breastmilk consumption on the day of the food record. On average, breastfed toddlers consumed fewer servings of the five food groups, including dairy. While breastfeeding into the second-year benefits mother and child [18], too much breastmilk may displace the intake of other nutrient-rich foods [19]. There is a need for practical advice in the infant feeding guidelines for parents on balancing breastfeeding with the intake of other foods. 

Discretionary foods contributed around 12% of the total energy intake of toddlers. Specific advice for parents on encouraging healthy snacking habits in their toddlers may prevent excessive consumption of snacks classified as discretionary foods. In older toddlers, discretionary foods accounted for up to one-quarter of energy intake, consumed primarily as snacks.

### 2.4. Nutrient Intakes

OzFITS 2021 was the first Australian nationwide study to estimate nutrient intakes for children less than 2 years [20]. For infants under 12 months, we could not determine the prevalence of inadequacy for most nutrients, as there is no Estimated Average Requirement (EAR), only an Adequate Intake (AI). An intake below the AI does not indicate inadequacy for a nutrient; however, population intakes above the AI suggest nutritional adequacy. Most nutrient intakes exceeded the AI for infants. 

Only two nutrients have established EARs for infants 7–12 months, iron, and zinc, both of which had a high prevalence of inadequacy at 92% and 17%, respectively. For toddlers, most nutrients have EARs, and the prevalence of inadequacy was less than 10% for all but iron, where 25% were below the EAR. 

### 2.5. Iron Intakes

We were surprised by the high prevalence of inadequate iron intake, especially in older infants. In the US FITS, the prevalence of inadequacy for iron in older infants was only 18%, likely due to the higher use of iron-fortified breast milk substitutes and iron-fortified cereal use in the US population [21,22]. In our study, infant formula and iron-fortified infant cereals were top sources of iron for infants, although less than one third consumed these products. Heme sources of iron, such as pureed red meat, were consumed by only 25% of infants and contributed to less than 5% of total iron. The EAR for iron (7 mg/day) may be too high in older infants and needs to be reconsidered. If not, it will be challenging to meet the EAR in breastfed infants without supplementation or fortified foods. 

### 2.6. Sodium Intakes

Excessive sodium intake was of concern, with 1 in 3 toddlers exceeding the upper limit of 1000 mg/day, the equivalent of 2.5 g of salt. Higher sodium intakes are correlated with blood pressure in children [23] and blood pressure tracks with age [24]. Interestingly, discretionary foods account for only 27% of sodium intake, whereas cereals and dairy account for 40%. Reducing discretionary food consumption will help reduce sodium intake, but other strategies are needed. Given high sodium intake is a problem across the Australian population, foods such as bread and cheese may need to be reformulated to reduce their salt content [25,26]. Under its Healthy Food Partnership, the Australian Government is working with industry to reformulate foods to enable manufacturers to provide healthier choices [26]. This reformulation includes sodium reduction targets for specific food categories.

## 3. Other Findings

### 3.1. Pouches

Since the Infant Feeding Guidelines were published in 2013, there has been a marked increase in commercial infant and toddler foods packaged in pouches with a spout [27]. These foods contain high amounts of sugar in the form of fruit puree, are poor sources of iron, and lack diversity in taste and texture [27]. In OzFITS 2021, 50% of children consumed food from pouches on the day of the food record. 

Although many manufacturers provide instructions to transfer the food from the pouch to a bowl and feed via a spoon, of the children consuming pouch foods, one-half sucked these foods directly from the spout. Sucking foods directly from a spout may delay the acquisition of oral feeding skills necessary to manage foods of varying textures. In addition, as chewing is essential for signaling satiety, there may be a risk of appetite dysregulation and obesity [28]. In OzFITS 2021, we identified a trend of foods offered to toddlers as drinks. Several parents/caregivers used refillable pouches or feeding bottles designed to pump food into baby’s mouths (Subo bottles^TM^) to feed homemade foods.

### 3.2. Juice and Smoothies

Unlike the United States, juice consumption is not an issue in Australia. Australian public health messaging encouraging water or milk as the main drink has been strong, and in the OzFITS 2021 cohort, only 2% of toddlers consumed juice [7,17]. Although juice consumption was low, many parents gave their child energy-dense, homemade fruit smoothies. Like juices, smoothies have the potential to alter appetite regulation and displace intake of other food, including whole fresh fruit.

### 3.3. Alternative Diets

Some dietary practices have the potential to limit nutritional intake, and parents/caregivers were asked if their child followed a special diet. Less than 3% of the parents in OzFITS 2021 indicated that their child followed a vegetarian or vegan diet. No parents reported following any other type of diet.

### 3.4. Impact of the COVID-19 Global Pandemic

Recruitment for OzFITS 2021 commenced during the SARS-CoV-2 global pandemic in early 2020. During this period, parents worked from home, and many children could not attend childcare. Although short-lived in most Australian states and territories, Victoria and New South Wales were under stay-at-home orders for 180 and 107 days, respectively. When asked if the pandemic influenced how they fed their children, (70/101) 69% of the parents that indicated an impact resided in Victoria and New South Wales. Although the responses varied, some common threads appeared. Most parents reported positive changes to feeding practices, including a resurgence of family mealtimes, more time to prepare home-cooked foods, less takeaway, and prolonged breastfeeding. 

## 4. Gaps in Evidence and Areas for Future Research

### 4.1. Need for National Representative Survey of 0-2-Year-Olds

OzFITS 2021 used convenience sampling and is not representative of the whole population. Our sample is more highly educated and of higher socio-economic status than the general population. We encourage the Australian Government to include infants and toddlers in their population-representative national surveys. Even if our sample was population representative, it would not provide reliable estimates of diet and nutrition in vulnerable groups. Parallel studies focused on Indigenous and culturally and linguistically diverse Australians are needed. As a starting point, our Aboriginal Feeding Infant and Toddler Feeding study attempts to fill this knowledge gap by enrolling Aboriginal families in urban, rural, and remote South Australia. 

### 4.2. Estimating Breastmilk Intake and Nutrient Composition

Our estimation of breastmilk intakes was based on equations derived from a small number of exclusively or predominantly breastfed infants before and after feeding and validation in older infants through doubly labeled water [29,30]. This method does not account for differences in consumption patterns for mixed-fed infants, nor does it reflect the variation in feeding efficiency between children. Moreover, test-weighing before and after each food may interrupt the natural rhythm of breastfeeding and infant-mother interaction [31]. Finally, this method has not been validated in toddlers. More robust equations are required based on doubly labeled water and a larger sample of infants and toddlers. 

For breastfed infants and toddlers, we relied on breastmilk nutrient composition from the 2011/13 Australian Food, Supplement, and Nutrient Database [32,33]. However, most of these values were adopted from the US and were developed almost 30 years ago. The maternal diet can alter breastmilk composition, particularly fatty acid and lipid profiles [34]. Moreover, many women in Australia take a micronutrient supplement during pregnancy and lactation that will increase breastmilk concentration of some nutrients such as iodine, thiamine, and folate [32]. Australian health authorities recommend that mothers take an iodine supplement during pregnancy to increase breastmilk iodine concentration, so we have likely under-estimated iodine intakes of the children enrolled in OzFITS 2021. There is a need to update food composition tables for breastmilk to reflect changes in food consumption and supplement use.

### 4.3. Inclusion of Breastmilk in Food-Based Dietary Guidelines 

The Food-Based Dietary Guidelines give the suggested number of servings and serving sizes for each food group required to meet the energy and nutrient needs of each sex and life stage group [15,16]. For toddlers, the challenge in developing the guidelines was to meet their high nutrient requirements in a limited amount of food. Breastmilk was not included in the modeling that informed the guidelines. In our study, over 40% of toddlers were breastfed. While breastfeeding benefits the toddler, breastmilk will displace other food groups. Breastmilk is iron poor and has half as much calcium as cow’s milk, despite having a similar energy content [7,32]. Modeling is needed that includes breastmilk to determine how it can be incorporated with other food groups and still meet toddler nutrient requirements. 

### 4.4. Improved Nutrient Reference Values for Infants and Young Children

Establishing evidence-based EARs for this age group will be challenging, especially those based on experimental studies. For infants under 12 months, a lack of EARs for nearly all nutrients precludes us from estimating the prevalence of inadequacy for this group. Further, most AIs and EARs were adopted from US Dietary Reference Values established as early as the mid-1990s and were developed with limited evidence. The AIs for infants under 12 months are often based on the nutrient composition of breastmilk from a small number of lactating women. Similarly, the EARs for young children were extrapolated from AIs or other age groups and adjusted for body size due to a lack of experimental evidence. 

### 4.5. The Need to Measure Nutrient Biomarkers in Infants and Toddlers

There is an urgent need to measure biomarkers to confirm our findings. Parents are understandably reluctant to allow their young children to undergo potentially painful blood draws for research purposes, and we need less invasive biomarkers. Reference ranges and cutoffs for nutritional biomarkers need to be developed for this age group. Urinary markers or dried blood spot methods requiring only a heel prick to collect blood are needed.

The need to measure biomarkers is most pressing for iron. Over 90% of older infants in OzFITS 2021 had inadequate iron intakes; however, we have no population-representative data on the prevalence of iron deficiency in young children. Before implementing iron supplementation or fortification strategies, we need confirmation of iron deficiency by measuring iron biomarkers and hemoglobin in this population.

## 5. Conclusions

**Figure 1:**
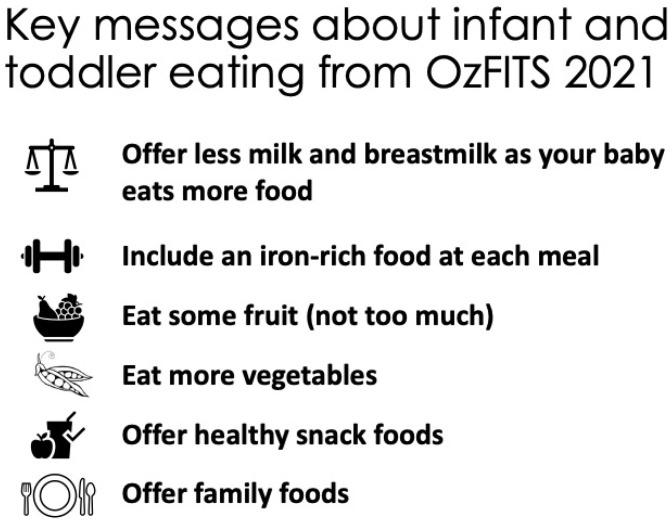
Key Messages.

OzFITS 2021 provides essential baseline data on infant and young child feeding in Australia and has allowed us to identify where things are going well and areas that need improvement. The majority of infants were breastfed, and many were breastfed into the second year. Most infants were introduced to solid foods around 6 months of age, including common allergens by one year. While most children consumed adequate amounts of most nutrients, the prevalence of inadequate iron intake was of concern, as was their high sodium intake. Key messages from the study are found in Figure 1. The findings from OzFITS 2021 are timely, as the Australian Dietary Guidelines are currently being revised. Ultimately OzFITS 2021 will inform the need for interventions to improve young child feeding practices in Australia.

## Data Availability

The data presented in this study are available on request from the corresponding author.
